# Supplementary material for: Spin switchable optical phenomena in Rashba band structures through intersystem crossing in momentum space in solution-processing 2D-superlattice perovskite film
Source: Nat Commun. 2025 May 7;16:4247. doi: 10.1038/s41467-025-57834-2 (PMC12059043; doi:10.1038/s41467-025-57834-2)
Supplement: Supplementary file 1 — Supplementary Information [file 41467_2025_57834_MOESM1_ESM.pdf]

Supporting Information

Spin Switchable Optical Phenomena in Rashba Band Structures through Intersystem Crossing in Momentum Space in Solution-Processing 2D-Superlattice Perovskite Film

Bogdan Dryzhakov[1], Yipeng Tang[1], Jong Keum[2,3], Haile Ambaye[2], Jinwoo Kim[4], Tae-Woo Lee[4], Valeria Lauter[2*], Bin Hu[1*]

[1] Department of Materials Science and Engineering, University of Tennessee, Knoxville, TN 37996, USA
[2] Neutron Scattering Division, Neutron Sciences Directorate, Oak Ridge National Laboratory, Oak Ridge, 37831, Tennessee, USA
[3] Center for Nanophase Materials Sciences, Oak Ridge, TN, 37831, Tennessee, USA; Neutron Scattering Division, Oak Ridge, TN, 37831, Tennessee, USA
[4] Department of Materials Science and Engineering, Seoul National University, Seoul, 08826, Republic of Korea

[*]Corresponding author.
E-mail address:
lauterv@ornl.gov
bhu@utk.edu

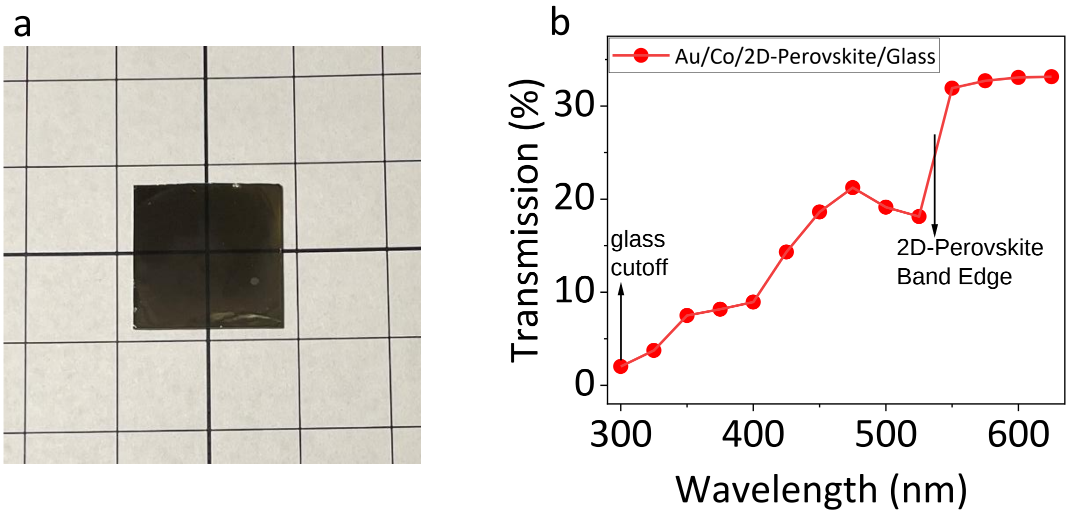

Figure S1: (a) Optical photo under white light of transparent semiconductor/metal thin films on glass substrate (Au/Co/2D-perovskite/Glass). (b) Transmission curve of the thin film. The measurement uses a white light Xenon lamp, whose light is filtered into a narrow band using gratings. The power transmitted at each wavelength, with respect to a blank, is measured using a power meter and calculated as $T(\%) = (P_{blank}-P_{film})/P_{blank}$. Glass absorption is significant around 300 nm, and the hybrid perovskite sharply absorbs at the 525 nm band-edge. The combined 17 nm Co and 12 nm Au layers are semi-transparent, allowing for 20% to 30% light transmission around the photoluminescence (PL) peak.

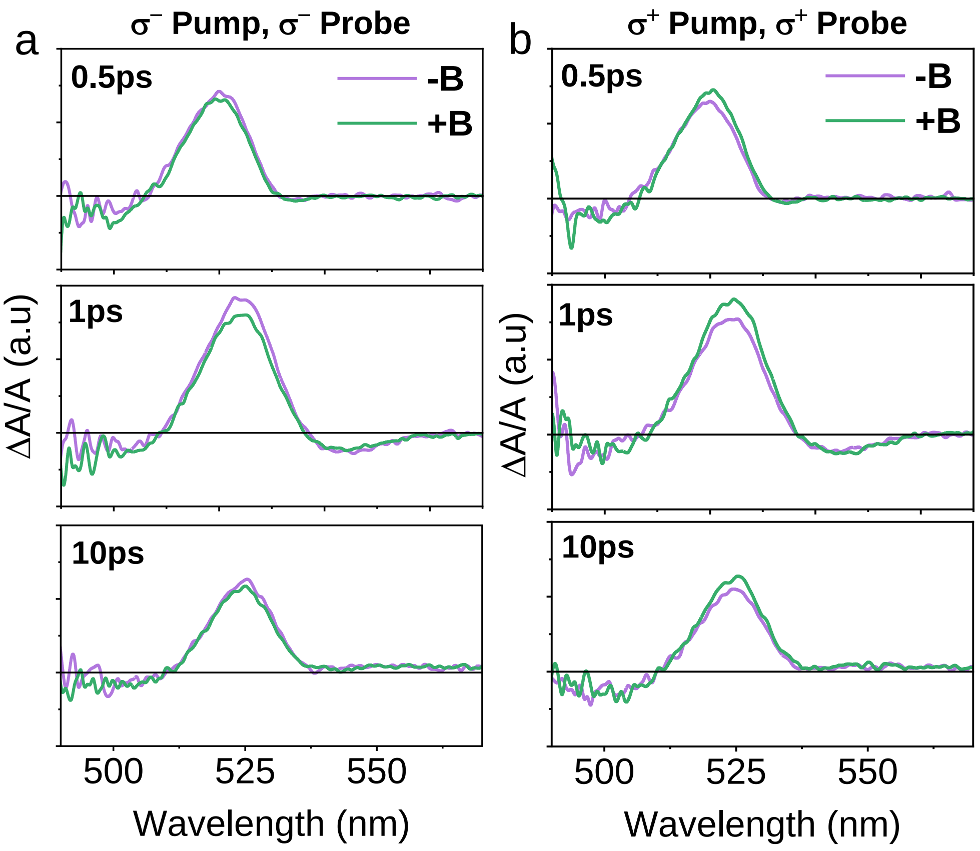

Figure S2: Transient absorption spectra at selected early absorption times (0.5, 1, 10 ps) with co-polarized pump and probe beams (σ^+ or σ^-) under applied magnetic field (+B or -B). Photogenerated carriers in 2D-perovskite are modified by the spin of Cobalt, aligned by an external magnetic field, leading to changes in polarization-sensitive photoinduced absorption intensity.

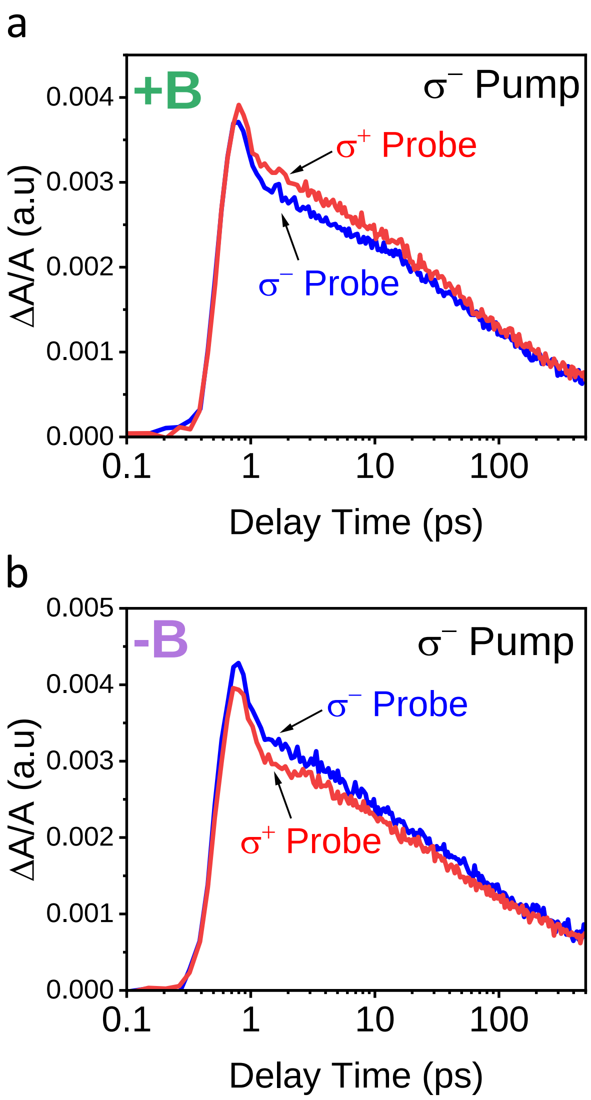

Figure S3: Transient absorption spectral decay of the photoinduced absorption feature when using σ^- pump and co- or cross-polarized probe beams under an applied magnetic field with opposite directions (a) +B and (b) -B. Spin selectivity, induced through spin-Rashba interactions between the Co and 2D-superlattice hybrid perovskite, occurs rapidly within the early-time dynamics dominated by Rashba-type interactions.

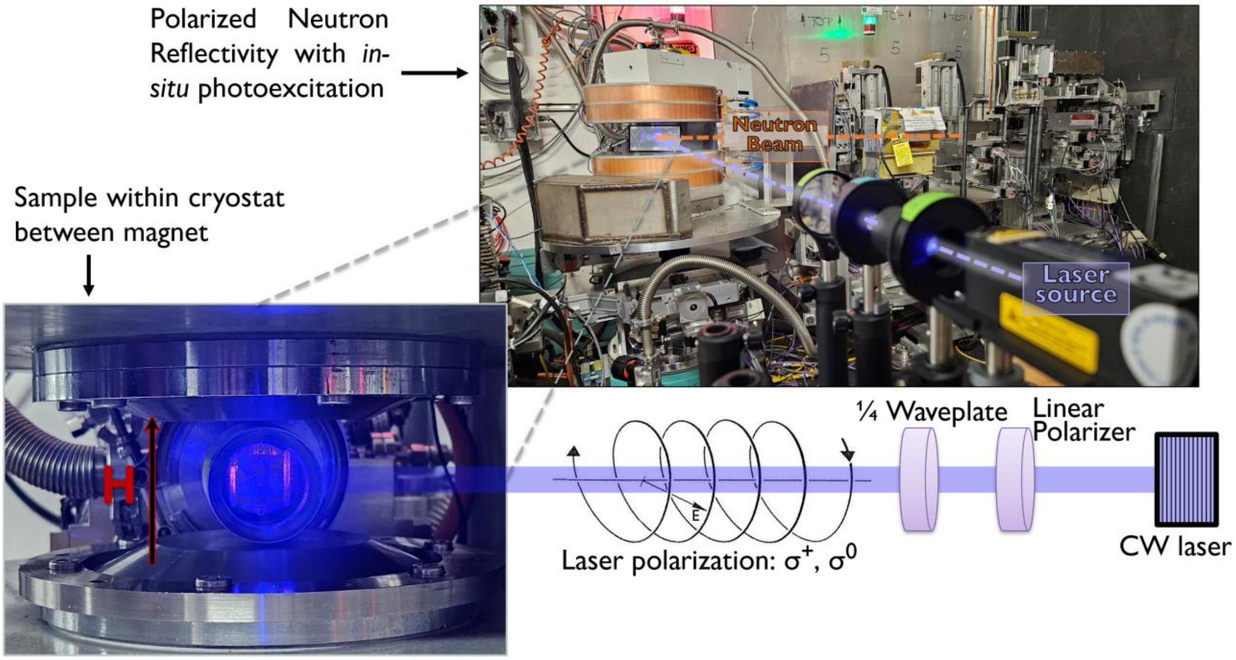

Figure S4: Polarized neutron reflectometry with *in-situ* polarized photoexcitation experimental setup. The circularly polarized 405 nm continuous-wave laser is expanded onto the thin film sample, which sits inside a cryostat with sapphire optical window. Cobalt layer spin is aligned with an in-plane external magnet field. A polarized neutron beam is reflected carrying both magnetic and nuclear scattering information corresponding to depth-dependent magnetization.

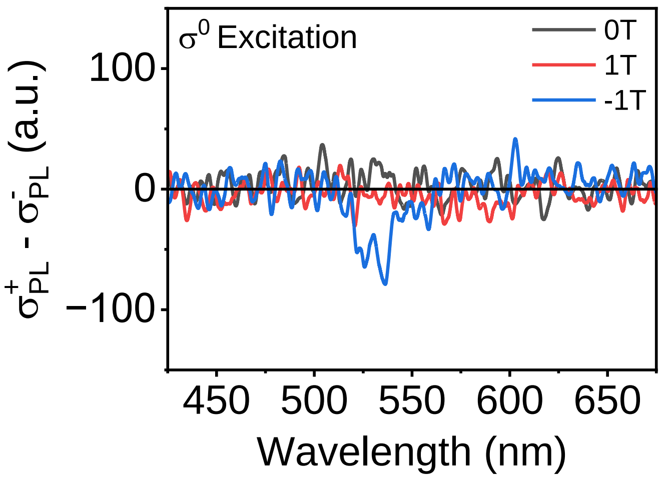

Figure S5: Circular polarization resolved photoluminescence under a 343nm linearly polarized excitation (σ^0) and magnetic field conditions (0T, -1T, 1T). Co/2D-superlattice thin films without circularly polarized photoexcited spin-orbital interactions exhibit negligible signal of circular polarized light emission under the three magnetic field conditions switching the cobalt magnetization condition.

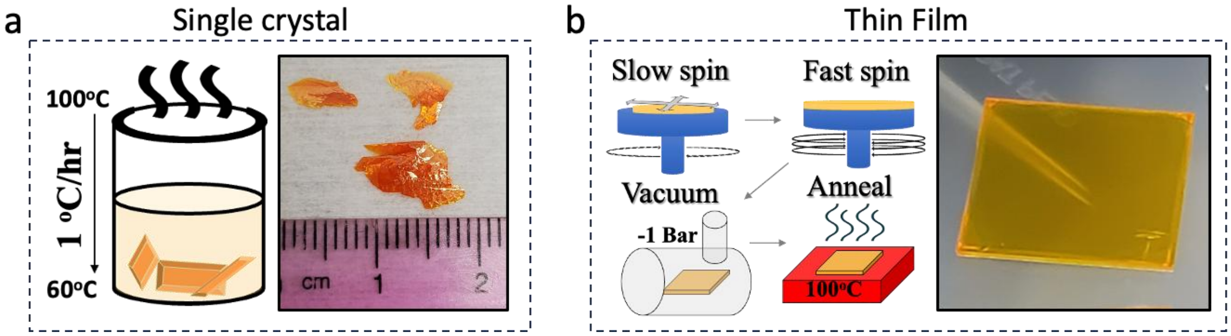

Figure S6: Materials synthesis and preparation schematics. (a) Single crystal growth through temperature cooling, showing the resulting centimeter-sized, orange crystals. (b) Thin film fabrication process via spin-coating, including slow and fast spin steps, vacuum treatment, and thermal annealing, with the final vibrant colored thin film product shown.

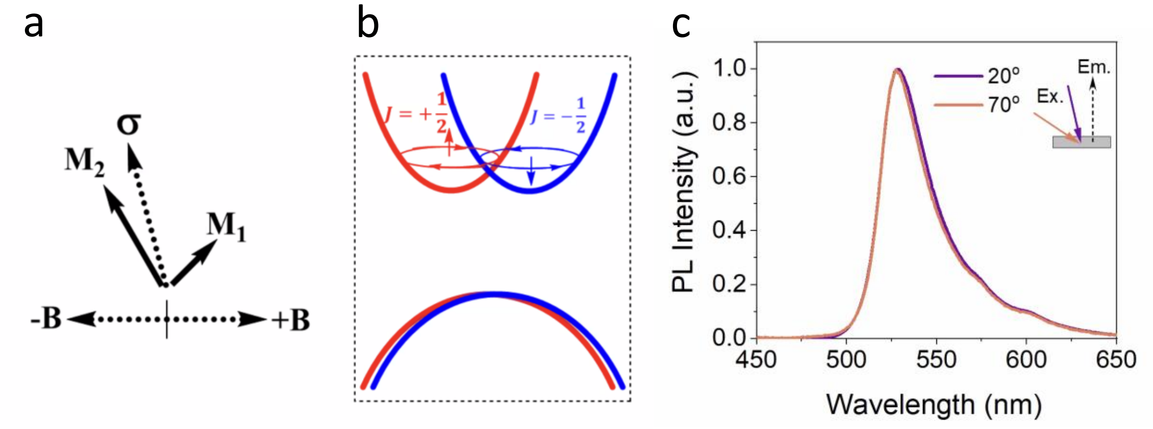

Figure S7: (a) σ is the magnetic dipole from circularly-polarized orbital. M_1 and M_2 are optically induced magnetizations at +B and -B, respectively. The optically induced magnetizations, M_1 and M_2, arise from this interaction and have magnitudes that depend on the direction of the applied magnetic field: smaller M_1 under a positive magnetic field (+B) and larger M_2 under a negative magnetic field (-B). (b) Rashba band structure with spin-up and spin-down bands and the intersystem crossing between these bands under different magnetic field directions. Under a negative magnetic field (-1 T), a larger optically induced magnetization enhances intersystem crossing between spin-up and spin-down bands in the Rashba band structure, leading to differences in population dynamics for σ^+ and σ^- excitations. Conversely, a positive magnetic field (+1 T) results in a smaller optically induced magnetization, causing negligible intersystem crossing and similar population dynamics for both spin polarizations. This variation in intersystem crossing with magnetic field direction provides further experimental evidence supporting the presence of Rashba band structures in our 2D-superlattice perovskite film. (c) Normalized photoluminescence spectra at different incident angles of excitation beam.

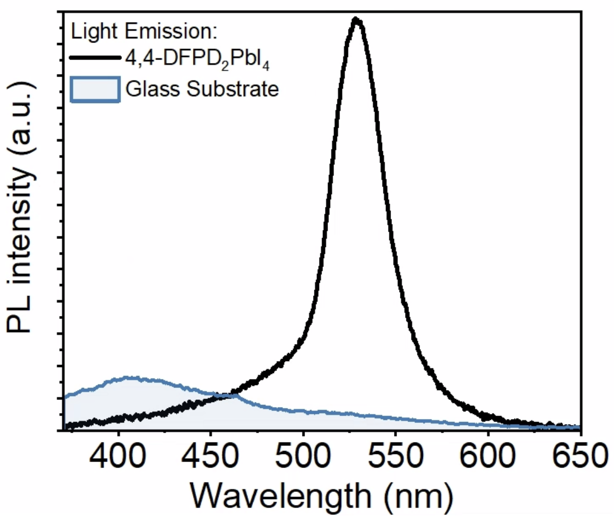

Figure S8: Reference emission spectra of the amorphous glass substrate under 343 nm laser excitation is overlayed on the 4,4-DFPD$_2$PbI$_4$ PL. Emission from the glass appears as a high-energy shoulder in the room-temperature spectra shown in Figures 2d, 2e, 3b, and 3c.
